# The Different Fate of the Pyrenean Desman (*Galemys pyrenaicus*) and the Eurasian Otter (*Lutra lutra*) under Climate and Land Use Changes

**DOI:** 10.3390/ani13020274

**Published:** 2023-01-13

**Authors:** Luca Francesco Russo, Ángel Fernández-González, Vincenzo Penteriani, María del Mar Delgado, Santiago Palazón, Anna Loy, Mirko Di Febbraro

**Affiliations:** 1 EnvixLab, Department of Biosciences and Territory, Università degli Studi del Molise, 86090 Pesche, Italy; 2 BIOSFERA Consultoría Medioambiental S.L., C/Candamo n°5., 33012 Oviedo, Spain; 3 National Museum of Natural Sciences (MNCN), Department of Evolutionary Ecology, Spanish National Research Council (CSIC), c/José Gutiérrez Abascal 2, 28006 Madrid, Spain; 4 Biodiversity Research Institute (IMIB), CSIC/UO/PA, Campus de Mieres, Edificio de Investigación, 33600 Mieres, Spain; 5 Direcció General de Polítiques Ambientals i Medi Natural, Generalitat of Catalonia, Carrer del Foc, 57, 08038 Barcelona, Spain; 6 Grupo Nutria, Sociedad Española Para la Conservación y Estudio de los Mamíferos (SECEM), 29080 Málaga, Spain

**Keywords:** predator–prey interactions, *Lutra lutra*, *Galemys pyrenaicus*, climate change, land use change, species distribution models

## Abstract

**Simple Summary:**

In the last century, hundreds of species have gone extinct or have undergone a decline in population size due to climate and land use change. Species can respond to these alterations in different ways, with some species facing extinction while others benefit from these changes. Here, we investigate the effects of climate and land use change on two semi-aquatic mammals, the Pyrenean desman (*Galemys pyrenaicus*) and the Eurasian otter (*Lutra lutra*), and how climate and land use change might affect the interaction between these two species. We found that the otters will take advantage of these environmental alterations, while desmans will undergo a drastic reduction of their suitable habitats. In addition, the availability of overlapped range margins between the two species might increase, exposing desmans to a potential increased predation risk by otters.

**Abstract:**

Climate and land use change can affect biodiversity in different ways, e.g., determining habitat loss, altering reproduction periods or disrupting biotic interactions. Here, we investigate the effects of climate and land use change on the spatial distribution of two semi-aquatic mammals, the Pyrenean desman (*Galemys pyrenaicus*) and the Eurasian otter (*Lutra lutra*). We first modeled the current potential distribution of the desman and the otter in the Iberian Peninsula, considering topographic, climatic and land use variables. Second, we predicted their potential distribution in 2050 under climate and land use change scenarios. We calculated the percentage of range gain/loss and shift predicted for the two species under such scenarios and quantified the present and future spatial overlap between the two species distribution. Irrespective of the scenario, desman models show loss of suitable habitat, whereas the otter will undergo an opposite trend. Aside from a preponderant habitat loss, the desman is predicted to increase its spatial overlap with otter range under the optimistic scenarios, potentially meaning it will face an exacerbated predation by otters. The potential increase of both habitat loss and otters’ predation might represent a major threat for the desman, which may affect the long-term persistence of this endemic species in the Iberian Peninsula.

## 1. Introduction

In the last century, hundreds of species have gone extinct or have undergone a decline in population size due to global change drivers [1,2], with endangered and endemic species being the most threatened due to their small range of distribution and numbers [3]. However, species can respond in different ways to the alterations posed by climate and land use change, with some species facing extinction while others benefit from these changes [1,4,5]. Increasing temperatures, reduced precipitations and alterations in land use will entail a decline in habitat availability [6]. In response to these alterations, species can shift their distribution, adapt to the new environment or decline to extinction [7,8,9,10].

Climate and land use changes can also lead to an alteration of species interactions [11]. For example, the alterations in prey–predator interactions can influence the likelihood of encounter, mortality and predation risk [12,13]. Changes in the availability and distribution of the prey might also cause a shift in the predator diet and impact different species [14]. For example: in the Nepalese Himalayas, climate change could alter the niche overlap between the snow leopard (*Panthera uncia*) and the blue sheep (*Pseudois nayaur*), hampering the snow leopard’s availability of its preferred prey [15]; Bastille-Rousseau et al. [16] found that future climate conditions could increase the predation of caribou (*Rangifer tarandus*) by the coyote and decrease the predation of caribou by the black bear (*Ursus americanus*); and Penteriani et al. [17] showed that future climate change scenarios will reduce the distribution of seven plant species that are part of the diet of brown bears (*Ursus arctos*), resulting in a reduction in this species distribution and a shifting towards a more carnivorous diet.

In this context, it is particularly relevant to assess how climate and land use change may affect the availability of environmental conditions where the predator–prey interaction can occur, i.e., the modifications of spatial overlap between prey and predator species under future scenarios. For instance, a prey species might undergo, in parallel, a reduction in its distribution and an increase in spatial overlap with occasional predators as a consequence of global change habitat alterations, thus suffering from multiple threats at the same time. Having set this scenario, we investigated the effects of climate and land use changes on the potential predator–prey interaction of two semi-aquatic mammals, the Pyrenean desman (*Galemys pyrenaicus*, hereafter desman) and the Eurasian otter (*Lutra lutra*, hereafter otter) in the Iberian Peninsula. The desman is a small mammal of the *Talpidae* family, occurring only in the Pyrenees, northern and central Spain and northern Portugal [18]. As a semi-aquatic mammal that prefers fast-flowing streams, this species has suffered a serious decline in recent years due to habitat loss and fragmentation, waterways pollution, persecution by fishermen and introduction of invasive species [18]. It is classified as *Endangered* on the IUCN red list and listed on the EU Habitats and Species Directive (Annexes II and IV) and Bern Convention (Appendix II).

Furthermore, due to its specialist habits and low dispersal abilities, this species seems to be particularly vulnerable to global change, with a likely sizeable loss of suitable habitat even under the more optimistic future change scenarios [19,20]. The otter is a mammal of the *Mustelidae* family widely distributed in Eurasia and part of Africa [21] and is also considered a semi-aquatic mammal able to tolerate different types of aquatic environments [22]. The diet of this species is mainly composed of fish [23,24,25], although it can also prey on other species such as the desman [26,27,28].

The desman underwent a severe decline in the last century in most of Europe, caused by habitat fragmentation and loss, hunting for fur and pollution of waterways. However, following legal protection, habitat restoration and banning of harmful pollutants, the desman is now recovering in its former range [21]. The otter is considered as *Near Threatened* on the IUCN red list and listed in Appendix II of the Berne Convention, Annexes II and IV of the EU Habitat Directive (43/92/CEE) and Appendix I of CITES. Due to its environmental flexibility, this species seems not to be globally affected by climate and land use changes, with even predicted positive effects of climate change on habitat suitability [29,30]. Since it is known that desmans share their habitat [31,32] and are occasionally preyed [26,27,28] on by otters, we explored if and to what extent the above-mentioned changes in climate and land use change may determine an alteration of the spatial overlap between the two species distributions, and, hence, a potential increase in otter predation rates on desman.

To better understand how global change drivers could alter the geographic distribution of the potential otter–desman predation areas, we (i) modelled the current potential distribution of desman and otter in the Iberian Peninsula, considering topographic, climatic and land use variables; (ii) predicted their potential distribution in 2050 under climate and land use change scenarios explicitly accounting for their dispersal capabilities; (iii) calculated the percentage range of gain/loss and shift predicted for the two species under such scenarios; and (iv) quantified the present and future spatial overlap between the two species’ distribution.

## 2. Materials and Methods

### 2.1. Occurrence Data

We collected the desman presence data during seven campaigns of sampling held in Spain and Portugal from 2013 to 2018. Specifically, as desman scats are easy to recognize when fresh [33], we searched latrines through 200 m transects placed inside the hydrographic network. We also gathered 113 presence data of desman, with spatial uncertainty of less than 250 m radius, obtained from the “Mammals in Portugal” open source repository, which stores data of all the mammal species occurring in Portugal [34]. From both strategies, we collected 412 occurrence data for the desman from 2013 to 2020. Otter presence data were obtained from the Spanish National Otter Monitoring initiative [35]. In this case, we considered 5879 presence data, which had a spatial uncertainty of less than 250 m radius. Since citizen science data are often used to build suitability models, i.e., [36,37], we also retrieved 835 otter presence data from the iNaturalist portal (www.inaturalist.org (accessed on 20 June 2022)), as well as 301 data from the “Mammals in Portugal” repository [34]. Also for these data, we retained only the records with a spatial uncertainty of less than 250 m radius. In total, we collected 7015 otter occurrence data from 2000 to 2020.

**Figure A1:**
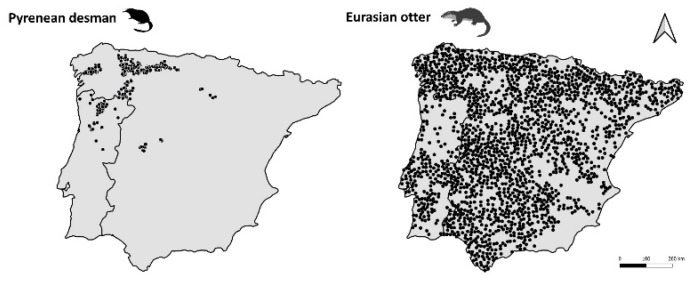
Distribution in the Iberian peninsula of the occurrence data of the desman (*n* = 251) from 2013 to 2020 and the otter (*n* = 1912) from 2000 to 2020, after the thinning procedure by the “spThin” R package [38].

To avoid spatial autocorrelation in occurrences data, we used the “spThin” R package [38], with thinning points laying closer than 20 km for otter and 2 km for the desman. We used these distances because they correspond to the dispersal distances of the two species [39,40,41]. After the thinning procedure, we retained 1912 otter occurrence records and 251 for desman (Figure A1).

### 2.2. Environmental Variables

We initially considered the 19 bioclimatic variables (Table A1) from the CHELSA database [42], rasterized at ca. 1 km spatial resolution. In addition, we included elevation [43], slope and roughness (the last two were calculated from the elevation map). We also considered the following six land use categories rasterized at ca. 1 km spatial resolution: forests, grasslands, farmlands, urban areas, water bodies and barren, gathered from Li et al. [44]. Specifically, each of the six categories was transformed from categorical to continuous by calculating the Euclidean distance from each pixel [45]. We focused on climate, land use and topographic variables as they are known to be important environmental drivers in freshwater ecosystems [46] and have already been considered in other studies describing both otter [30] and desman [19] habitat characteristics. Since the two study species are water-dependent mammals [18,22], all the eco-variables were clipped within a 1 km radius buffer around the hydrographic network derived from Schneider et al. [47]. To avoid correlation between the variables, we dropped predictors reporting a variance inflation factor > 5 [48,49]. After checking for multicollinearity, we retained the following 14 variables for SDM calibration: isothermality (BIO3), temperature seasonality (BIO4), mean temperature of wettest quarter (BIO8), mean temperature of driest quarter (BIO9), precipitation seasonality (BIO15), precipitation of coldest quarter (BIO19), slope, elevation and all the six land use categories.

### 2.3. Species Distribution Models

Relying on species occurrences and the above-mentioned environmental variables, we predicted otter and desman distribution by using an ‘ensemble forecasting approach’, as implemented in the “biomod2” R package [50]. Specifically, the following algorithms were used: generalized linear models (GLM), generalized additive models (GAM), generalized boosted models (GBM), random forest (RF) and Maxent. For each of the two species, we randomly placed 10,000 background points over an area identified by all the WWF Terrestrial Ecoregions [51] encompassing species records, according to the “BAM” framework by Barve et al. [52]. Since spatial partitioning cross-validation approaches to evaluate SDM predictive accuracy proved useful in assessing model transferability [53] and penalizing models based on biologically meaningless predictors [54], we used the “checkerboard2” strategy implemented in the “ENMeval” R package [55]. According to this strategy, data are partitioned into four binds along two hierarchical levels of spatial aggregation (further details are provided in Muscarella et al. [56]). For GLM, GAM, GBM and RF algorithms, we used the settings provided in Pio et al. [57] as a recommended configuration. For Maxent, we used the “ENMeval” package to test different setting configurations, so as to optimize the trade-off between goodness-of-fit and overfitting [56]. In particular, we tested regularization values between 0.5 and 4 (with 0.5 steps), along with the following feature classes: linear (L), linear + quadratic (LQ), hinge (H), linear + quadratic + hinge (LQH), linear + quadratic + hinge + product (LQHP) and linear + quadratic + hinge + product + threshold (LQHPT). Among the resulting 48 setting combinations, we chose the one reporting the lowest Akaike Information Criterion (AICc), a commonly used metric to perform model selection [58]. To evaluate SDM predictive performance, we used the area under the receiver operating characteristic curve (AUC; [59]) and the true skill statistic (TSS; [60]). Specifically, for AUC, prediction accuracy can be considered excellent (AUC > 0.90), good (0.80 > AUC < 0.90), fair (0.70 > AUC < 0.80) and poor (AUC < 0.60; [61]). As for TSS, prediction accuracy can be excellent (TSS > 0.75), good (0.40 > AUC < 0.75) and poor (TSS < 0.40; [62]). Moreover, only the models reporting AUC values ≥ 0.70 were considered in the subsequent analyses [63]. Ensemble models were obtained by calculating a weighted average of the individual model predictions with their AUC scores as weights [64].

### 2.4. Climate and Land Use Change Scenarios

All the models were projected over four climate and land use change scenarios forecasted to 2050. In particular, we considered the most optimistic (RCP2.6) and pessimistic (RCP8.5) climate change scenarios from the IPCC [65] in terms of the amount of greenhouse gas emissions. Specifically, these scenarios refer to Representative Concentrations Pathways (RCPs), with RCP2.6 indicating low greenhouse gas emissions, while RCP8.5 assumes a drastic increase in greenhouse gas emissions in the next decades. Climate change scenarios are elaborated from different meteorological research centers using models of the dynamics of physical components of the atmosphere and ocean circulation, called global circulation models (GCM). Since there is a certain variation in the GCM that can lead to differences in SDM projections [66], we used three alternative versions of the RCP scenarios [29], i.e., CCSM4, IPSL-CM5A-LR and MIROC-ESM-CHEM GCM. We selected these GCM since they provide non-redundant information about future climate modifications [67]. For land use change, we used a pessimistic scenario (A2) with a high alteration of land cover due to human population growth and a more optimistic scenario (B1) with low forecasted human population growth and reduced modification in land use [44]. By combining the four different forecasts, we obtained four possible scenarios in 2050, describing mild (RCP2.6 and LUC.B1), two intermediate (RCP2.6 and LUC.A2; RCP8.5 and LUC.B1) and extreme (RCP8.5 and LUC.A2) global change magnitude (further details are provided in Table A2). Since different binarization schemes can affect model results, we considered alternative thresholds [68]. Specifically, each model projection was binarized according to “equalize sensitivity and specificity”, “maximize TSS”, “mean occurrence probability” and “minimize receiver operating characteristic plot distance” thresholds.

When predicting future habitat suitability, we accounted for the ability of the studied species to disperse in future favorable environments. For this purpose, we used the “MigClim” R package [69], a cellular automaton model that takes into account species-specific restrictions in future projections in global change scenarios. Starting from current and future binary maps (0 = absence, 1 = presence), MigClim allows us to predict which cells might be colonized or decolonized in a given period, considering the limitations in dispersion such as the dispersal capacity of the species, reproductive maturity and potential barriers. Following Sales et al. [70], we considered SDM binary predictions clipped along the species IUCN range as the starting maps. Subsequently, we gathered information on reproductive age and dispersal capacity of the two species [39,40,41], considering 20 km for otter and 2 km for desman dispersal distance. Since the dispersion capacity of the desman is influenced by geographic basins and by the presence of dams [39], we included these two factors as barriers.

For each species and future scenarios, we applied two metrics of global change effects on the binary maps generated by MigClim, i.e., the net change and the geographical shift. Net change is calculated as the percentage that is gained/lost between current and future ranges (with respect to the stable portion), while the geographical shift was calculated as a percentage of overlap between current and future range maps [71]. Finally, for each threshold, period and scenario, we calculated the percentage of area overlap between otter and desman distribution.

## 3. Results

### 3.1. Species Distribution Models

**Figure 1:**
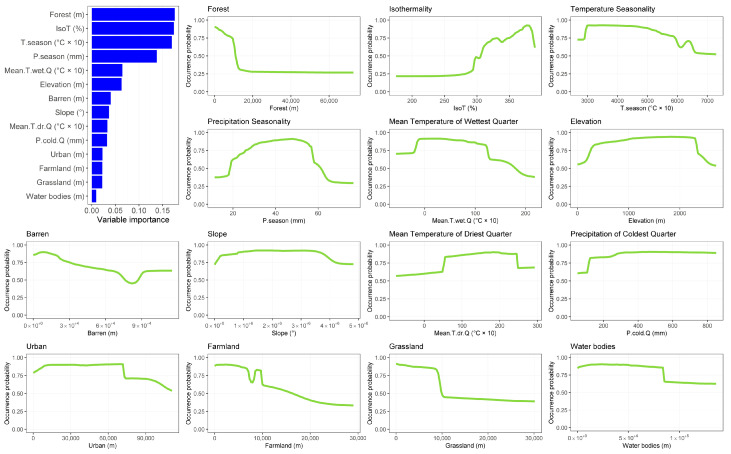
Bar plot depicting variables importance for desman and response curves depicting the shape of the relationship between desman habitat suitability and each environmental variable included into the ensemble model.

**Figure 2:**
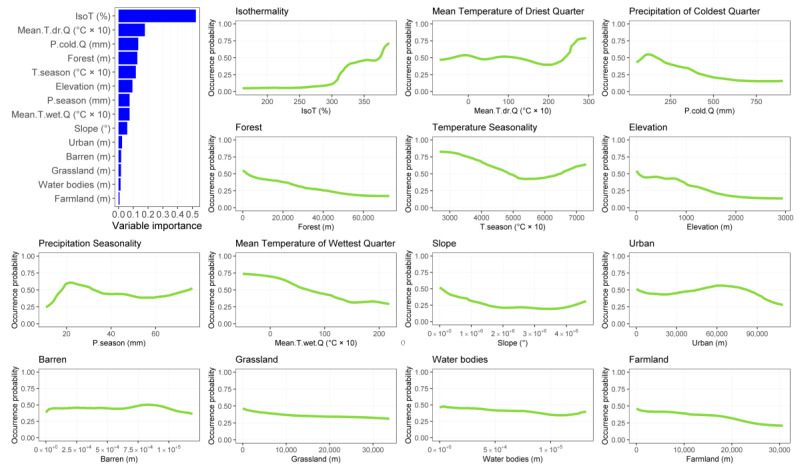
Bar plot depicting variables importance for otter and response curves depicting the shape of the relationship between otter habitat suitability and each environmental variable included into the ensemble model.

Desman and otter SDMs achieved good-to-excellent predictive performances sensu Swets [61], with the former species reporting a mean AUC = 0.98 (SD = ±0.003) and a mean TSS = 0.88 (SD = ±0.020) and the latter a mean AUC = 0.84 (SD = ±0.005) and a mean TSS = 0.52 (SD = ±0.010). Desman habitat suitability is mostly shaped by distance from forests, isothermality and seasonality in temperatures and precipitations (Figure 1). Specifically, the most suitable habitats for the species are close to forests and characterized by high isothermality values, low seasonality in temperatures and intermediate seasonality in precipitations (Figure 1). As for otters, isothermality has the most important effect on the species habitat suitability, along with mean temperature of driest quarter and distance from forests (Figure 2). In particular, the species found suitable habitats close to forests with high values of isothermality and temperature during the dry season (Figure 2).

### 3.2. Effect of Global Change Drivers on Species Distribution

**Figure 3:**
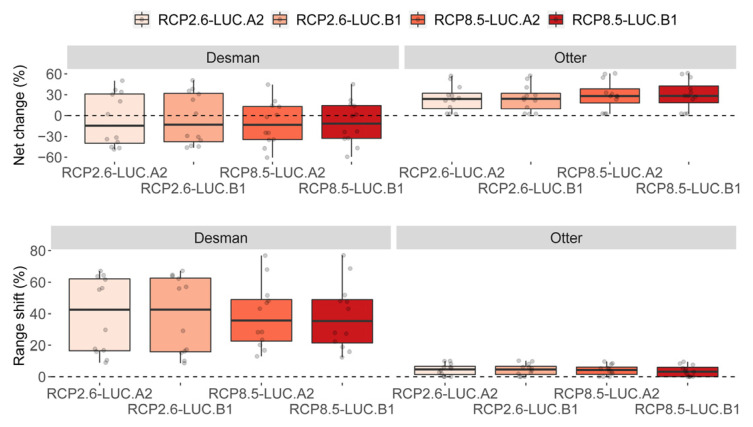
Percentage of range net change and shift for the desman and the otter under current time and the four 2050 global change scenarios. The variation depicted in each box plot refers to net change and shift values as generated by the three global circulation models and the four binarization thresholds.

**Figure 4:**
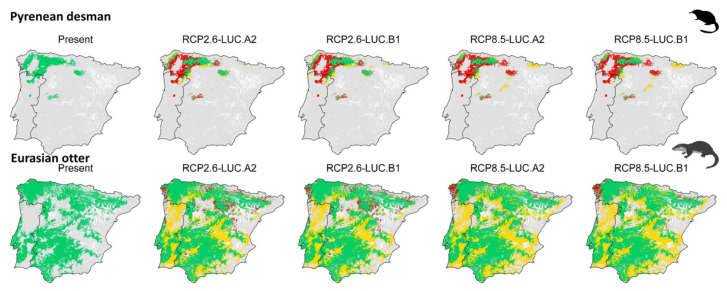
Spatially explicit predictions of desman and otter range modifications under 2050 climate and land use change scenarios, as generated by the IPSL-CM5A-LR global circulation model and the “equalize sensitivity and specificity” binarization threshold. Grey: stable unsuitable habitat, red: habitat loss, green: stable suitable habitat, yellow: habitat gain.

**Figure A2:**
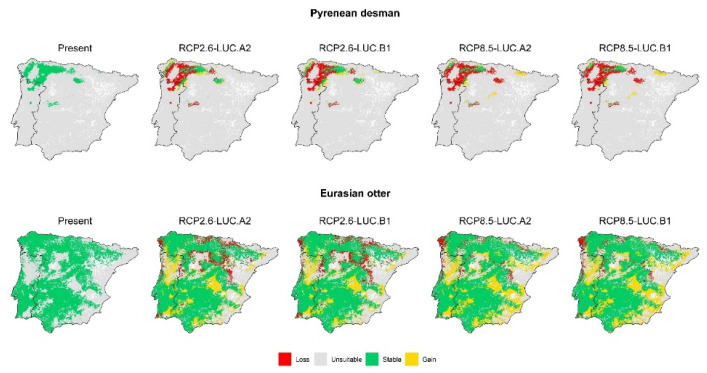
Spatially explicit predictions of desman and otter range modifications under 2050 climate and land use change scenarios, as generated by the IPSL-CM5A-LR global circulation model and the “maximize TSS” binarization threshold. Grey: stable unsuitable habitat, red: habitat loss, green: stable suitable habitat, yellow: habitat gain.

**Figure A3:**
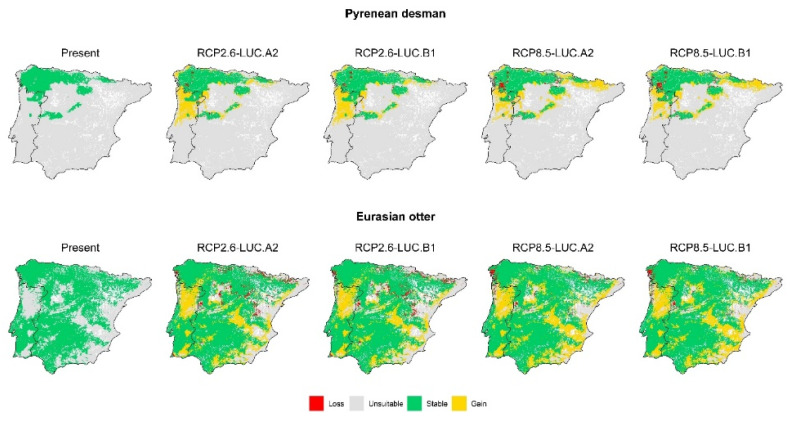
Spatially explicit predictions of desman and otter range modifications under 2050 climate and land use change scenarios, as generated by the IPSL-CM5A-LR global circulation model and the “mean occurrence probability”, binarization threshold. Grey: stable unsuitable habitat, red: habitat loss, green: stable suitable habitat, yellow: habitat gain.

**Figure A4:**
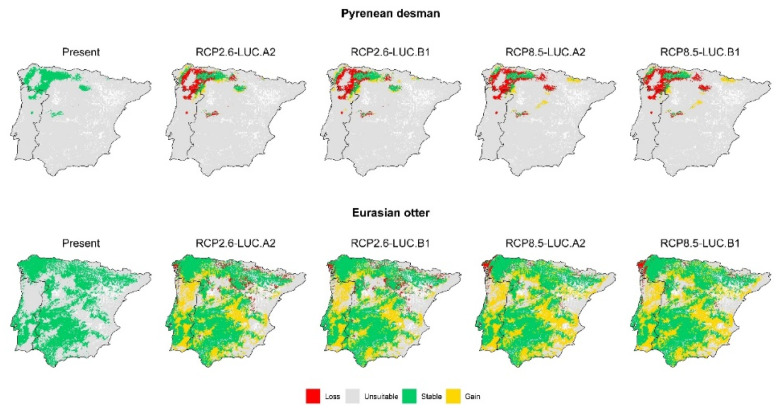
Spatially explicit predictions of desman and otter range modifications under 2050 climate and land use change scenarios, as generated by the IPSL-CM5A-LR global circulation model and the “equalize sensitivity and specificity”, binarization threshold. Grey: stable unsuitable habitat, red: habitat loss, green: stable suitable habitat, yellow: habitat gain.

**Figure A5:**
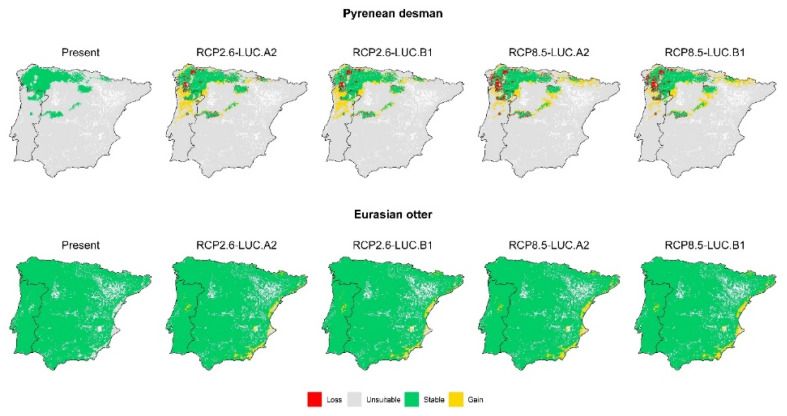
Spatially explicit predictions of desman and otter range modifications under 2050 climate and land use change scenarios, as generated by the IPSL-CM5A-LR global circulation model and the “minimize receiver operating characteristic plot distance”, binarization threshold. Grey: stable unsuitable habitat, red: habitat loss, green: stable suitable habitat, yellow: habitat gain.

**Figure A6:**
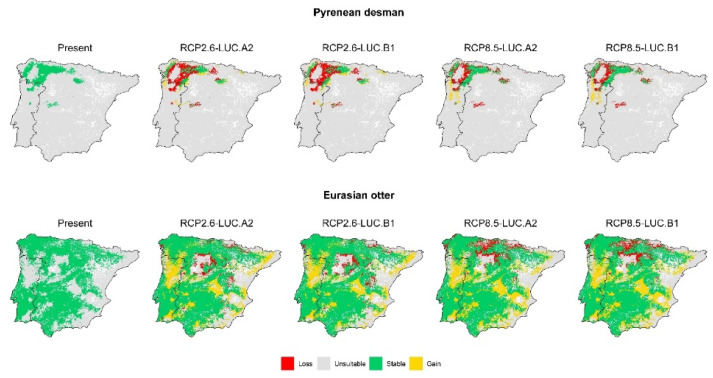
Spatially explicit predictions of desman and otter range modifications under 2050 climate and land use change scenarios, as generated by the CCSM4 global circulation model and the “maximize TSS” binarization threshold. Grey: stable unsuitable habitat, red: habitat loss green: stable suitable habitat, yellow: habitat gain.

**Figure A7:**
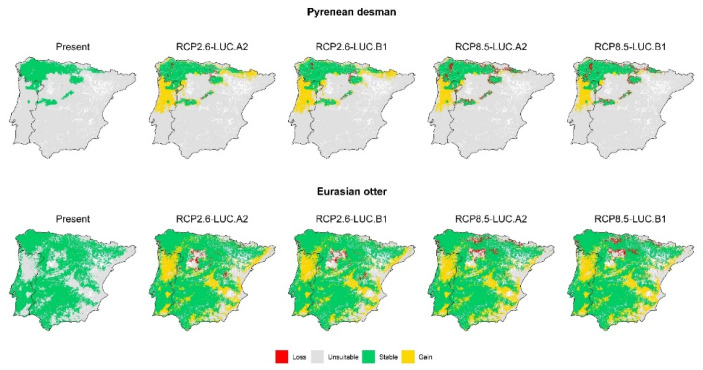
Spatially explicit predictions of desman and otter range modifications under 2050 climate and land use change scenarios, as generated by the CCSM4 global circulation model and the “mean occurrence probability”, binarization threshold. Grey: stable unsuitable habitat, red: habitat loss green: stable suitable habitat, yellow: habitat gain.

**Figure A8:**
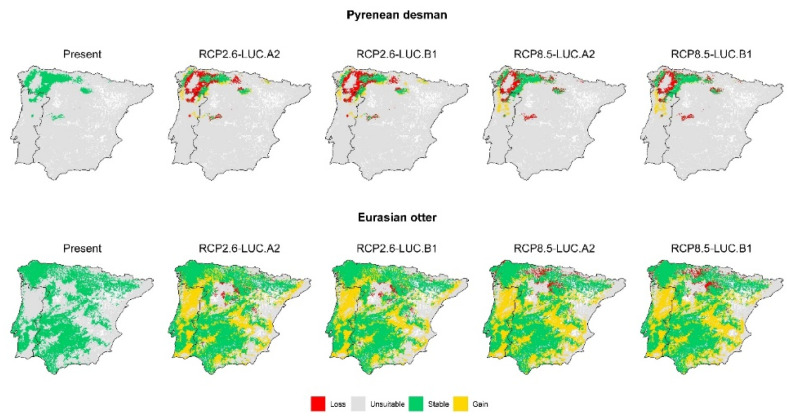
Spatially explicit predictions of desman and otter range modifications under 2050 climate and land use change scenarios, as generated by the CCSM4 global circulation model and the “equalize sensitivity and specificity”, binarization threshold. Grey: stable unsuitable habitat, red: habitat loss, green: stable suitable habitat, yellow: habitat gain.

**Figure A9:**
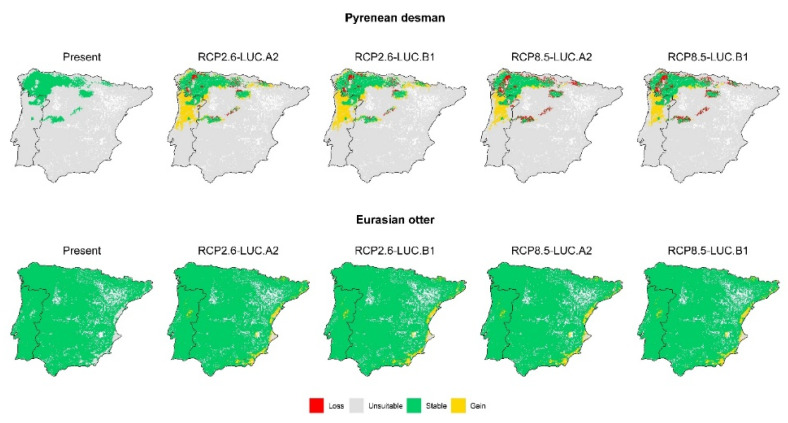
Spatially explicit predictions of desman and otter range modifications under 2050 climate and land use change scenarios, as generated by the CCSM4 global circulation model and the “minimize receiver operating characteristic plot distance”, binarization threshold. Grey: stable unsuitable habitat, red: habitat loss, green: stable suitable habitat, yellow: habitat gain.

**Figure A10:**
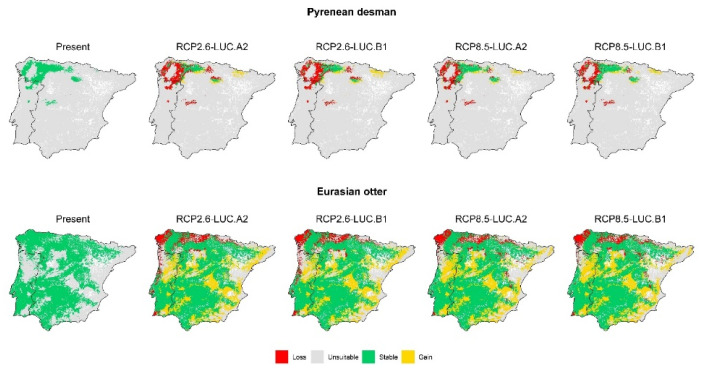
Spatially explicit predictions of desman and otter range modifications under 2050 climate and land use change scenarios, as generated by the MIROC-ESM-CHEM global circulation model and the “maximize TSS” binarization threshold. Grey: stable unsuitable habitat, red: habitat loss, green: stable suitable habitat, yellow: habitat gain.

**Figure A11:**
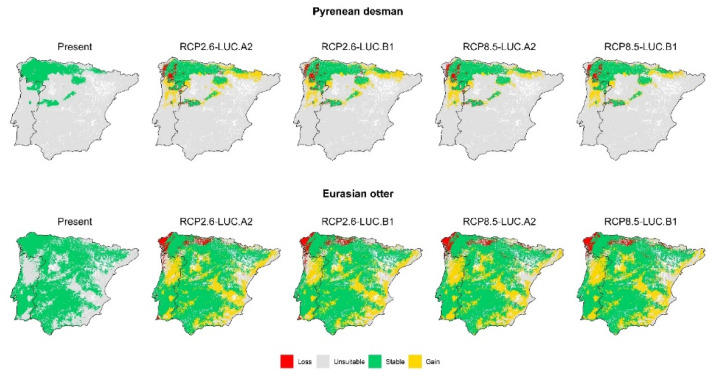
Spatially explicit predictions of desman and otter range modifications under 2050 climate and land use change scenarios, as generated by the MIROC-ESM-CHEM global circulation model and the “mean occurrence probability”, binarization threshold. Grey: stable unsuitable habitat, red: habitat loss, green: stable suitable habitat, yellow: habitat gain.

**Figure A12:**
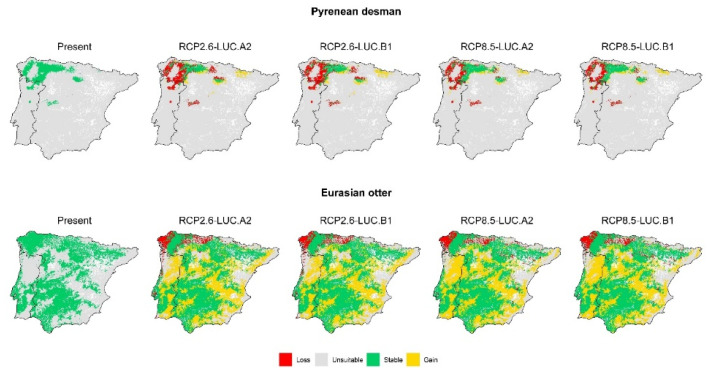
Spatially explicit predictions of desman and otter range modifications under 2050 climate and land use change scenarios, as generated by the MIROC-ESM-CHEM global circulation model and the “equalize sensitivity and specificity”, binarization threshold. Grey: stable unsuitable habitat, red: habitat loss, green: stable suitable habitat, yellow: habitat gain.

**Figure A13:**
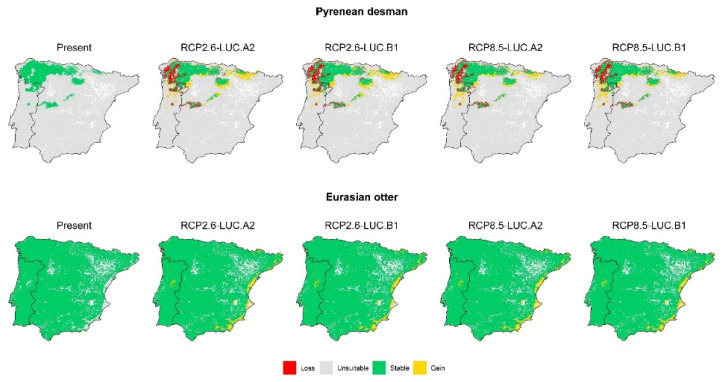
Spatially explicit predictions of desman and otter range modifications under 2050 climate and land use change scenarios, as generated by the MIROC-ESM-CHEM global circulation model and the “minimize receiver operating characteristic plot distance”, binarization threshold. Grey: stable unsuitable habitat, red: habitat loss, green: stable suitable habitat, yellow: habitat gain.

SDM predicted an overall detrimental effect on desman distribution under 2050 global changes, irrespective of the severity of the scenarios (Figure 3 and Figure 4). Under the optimistic scenarios (i.e., RCP.26–LUC.A2 and RCP.26–LUC.B1), desman distribution will likely undergo a ca. 15% reduction and a ca. 42% shift (Figure 3 and Figure 4; Figure A2, Figure A3, Figure A4, Figure A5, Figure A6, Figure A7, Figure A8, Figure A9, Figure A10, Figure A11Figure A12 and Figure A13) compared to its current range. The corresponding figure under the most pessimistic scenarios (i.e., RCP8.5–LUC.A2 and RCP8.5–LUC.B1) reported a negative range net change of ca. 15% and a range shift of ca. 35%. The otter showed a rather opposite pattern. In fact, both optimistic and pessimistic scenarios indicate a positive range net change (ca. 25%), while the shifted range percentage resulted in around 5% (Figure 3 and Figure 4; Figure A2, Figure A3, Figure A4, Figure A5, Figure A6, Figure A7, Figure A8, Figure A9, Figure A10, Figure A11Figure A12 and Figure A13).

### 3.3. Spatial Overlap

**Figure 5:**
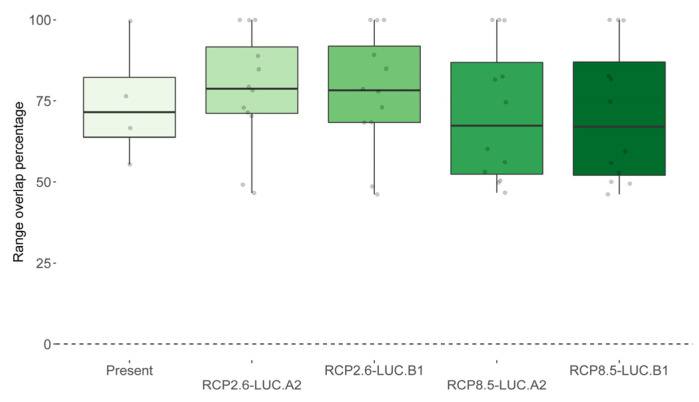
Percentage of overlap between the desman and otter ranges of distribution today and under the four 2050 global change scenarios. The variation depicted in each box plot refers to overlap values as generated by the three global circulation models and the four binarization thresholds.

The median percentage of desman distribution that overlaps with otter range under current environmental conditions is 71% (SD = ±19%; Figure 5). According to SDM predictions, this spatial overlap will undergo diverging patterns depending on climate and land use change scenarios. In fact, the percentage of desman distribution overlapping with otter range will likely increase above ca. 80% under the most optimistic scenarios (i.e., RCP2.6–LUC.A2, median = 79%, SD = ±18%; RCP2.6–LUC.B1, median = 78%, SD = ±18%), while decreasing below 67% under the most pessimistic ones (i.e., RCP8.5–LUC.A2, median = 67%, SD = ±21%; RCP8.5–LUC.B1, median = 67%, SD = ±21%; Figure 5).

## 4. Discussion

We found a clear difference in the response to climate and land use change between desman and otter, as well as a potential alteration of their predator–prey interactions. Specifically, we provided evidence that the desman will drastically reduce its distribution under 2050 climate and land use scenarios, whereas the otter will benefit from these environmental alterations in terms of gained range. Furthermore, our models predicted an increased overlapping between the desman and otter distribution range in 2050 under the most optimistic scenarios. This pattern could imply an increase in otter predation rates on desmans that, combined with the overall reduction in future distribution predicted for the desman, may pose a double threat for the long-term survival of this species.

The species distribution models for both the species indicated isothermality, i.e., day-to-night temperature oscillation, as one of the most important climatic variables that is positively related to species habitat suitability. Such a relationship has been previously found in other studies [72] and suggests that a more stable day-to-night temperature could allow semi-aquatic mammals, such as the desman and otter, to reduce energetic costs for maintaining a constant body temperature. The proximity to forested habitats also showed a certain importance in shaping habitat suitability for both species. In fact, it is well–known that riparian forests are important providers of cover for shelters and refuges [32,73,74]. Specifically for the desman, a low-to-intermediate seasonality in both temperature and precipitation represent a relevant driver influencing habitat suitability. These two variables are probably involved in characterizing more stable river conditions throughout the year, which are known to represent a key riverine habitat feature for the desman [75]. For the otter, the mean temperature of the driest quarter also emerged as directly related to habitat suitability. Some authors suggested that in Mediterranean ecosystems, the increase in temperature during the driest season determines a restriction of water flow and appearance of pools and ponds, triggering, in turn, an increase in otter prey abundance, e.g., crayfishes, cyprinids and amphibians [76,77].

The desman is predicted to lose a sizeable portion of its potential distribution range irrespective of the global change scenarios under consideration, which appears particularly concerning due to the desman’s scarce dispersal ability, capacity to cross artificial barriers and geographic basins of rivers [28,39]. In fact, we predicted the isolated population in the center of Spain to completely lose suitable habitat in 2050, similarly to the population from Portugal, with only small areas in North Spain remaining suitable. In addition to these large scale drivers, the desman is also threatened by small scale factors such as, e.g., habitat fragmentation and water pollution [18], with some local extinctions being already reported [75]. In light of that, we cannot exclude that even the habitat patches that remain suitable to 2050 might still be affected by the abovementioned small scale pressures, which were not accounted for in our predictions. As reported in several studies, the otter is predicted to take advantage of global change alterations forecasted to 2050 [30,45]. In line with this general evidence, we found that areas in the Iberian Peninsula that are now unsuitable for this species will become suitable under 2050 climate and land use changes, as a likely consequence of the predicted isothermality conditions. Such a prediction has been evidenced in both global [29,30] and local [45] scale investigations, and has been linked to the well-known high dispersal abilities of the species [41], which make it well capable to rapidly colonize new available areas [78,79,80].

Of great concern are the predictions we provided about the future dynamics of overlapping areas between desman and otter distributions. In fact, the desman range portion overlapping otter distribution is predicted to increase by ca. 7 to 8% in 2050 under the most optimistic scenarios (while decreasing under the most pessimistic ones). This pattern, coupled with the overall reduction in the entire specie range, would expose the desman to a worrying scenario in which the scarcer, residual distribution remnants in 2050 will become more suitable to otters than they are today, likely driving an increase in otter density. Given the well-described diet plasticity of otters [81], such a dynamic could even trigger a diet shift/widening, where the desman could go from being an occasional prey to a more frequent diet component, thus introducing an additional threat to the long-term conservation of this species. In fact, although the otter mainly feeds on fish [22], it can shift its diet in response to habitat, time of year, elevation and prey availability [82,83,84,85,86]. For instance, Cianfrani et al. [30] suggested that an increase in water temperature could cause eutrophication, with a drastic reduction in fish populations, and, consequently, in prey biomass for otters. This could trigger further shifting to alternative prey such as mammals and birds. That said, it is also plausible that such a diet shift could be rather limited due to the overall low densities of desman populations.

As for the most pessimistic scenarios, we reported a slight decrease in the overlap values, likely indicating that the extreme range reduction showed for desman under these scenarios will also involve most of the margins that were preserved and that overlapped with otter range under the most optimistic scenarios.

Among the main limitations of our research, we acknowledge that we did not account for possible future modifications of river status and dynamics triggered by global change. For example, we cannot exclude that the habitat patches remaining suitable in 2050 under climate and land use change might suffer from future hydrological stress or lower water availability. Such pressures could well make these patches unsuitable in terms of river health, especially for the desman, which prefers highly oxygenated water and rapid streams [87]. Moreover, our analysis focused strictly on the spatial overlap between desman and otters, and, therefore, only a potential increase in their interaction probability. In light of that, we acknowledge that multiple additional factors can contribute to predation events [88], such as, e.g., the density of both prey and predator populations [89,90] or small-scale habitat characteristics, such as the presence of human settlements or the availability of shelter sites [88]. Lastly, prey can change their use of space and circadian rhythm in response to the presence of a predator [91,92].

## 5. Conclusions

Our study highlighted how two semi-aquatic mammals can respond in very different ways to global change drivers. While otters will take advantage of these environmental alterations, likely continuing their increasing trend in the Iberian Peninsula [35], desmans will likely undergo a drastic reduction in their suitable habitats. In addition, under the most optimistic scenarios, the availability of overlapped range margins between the two species will likely increase, exposing desmans to a potential increased predation risk by otters. The synergistic pressure exerted by future habitat loss and increased predation rate pose a highly concerning scenario for the long-term conservation of this species. In light of this, we stress the importance of preserving riverine ecosystems against the effects of global change drivers, as well as implementing appropriate mitigation measures to ensure the persistence of this endemic species in the Iberian Peninsula.

## Data Availability

The data presented in this study are available on request from the corresponding author. The data are not publicly available due to ongoing research activities.

## Appendix A

**Table A1:** The considered 19 bioclimatic variables from the CHELSA database.

Codes	Names
BIO1	Annual Mean Temperature
BIO2	Mean Diurnal Range (Mean of monthly (max temp–min temp))
BIO3	Isothermality (BIO2/BIO7) (×100)
BIO4	Temperature Seasonality (standard deviation × 100)
BIO5	Max Temperature of Warmest Month
BIO6	Min Temperature of Coldest Month
BIO7	Temperature Annual Range (BIO5-BIO6)
BIO8	Mean Temperature of Wettest Quarter
BIO9	Mean Temperature of Driest Quarter
BIO10	Mean Temperature of Warmest Quarter
BIO11	Mean Temperature of Coldest Quarter
BIO12	Annual Precipitation

**Table A2:** Characteristics of 2050 climate and land-use scenarios used in SDMs projections.

Scenario	Main Characteristics
RCP.26	The scenario assumes that global annual CO_2_ emissions will peak between 2010–2020, declining substantially thereafter. A +1 °C global warming is forecasted [65]
RCP.85	The scenario assumes CO_2_ emissions keep rising throughout the 21th century. A +2 °C global warming is forecasted [65].
LUC.A2	The scenario assumes a high population growth, a sprawling urban expansion and a medium economic growth. As a result, the scenario predicts an increase in farmlands and a decrease in grasslands and forests [44].
LUC.B1	The scenario assumes a low population growth, a compact urban expansion and a high economic growth. As a result, the scenario predicts a decrease in farmlands and an increase in grasslands and forests [44].
